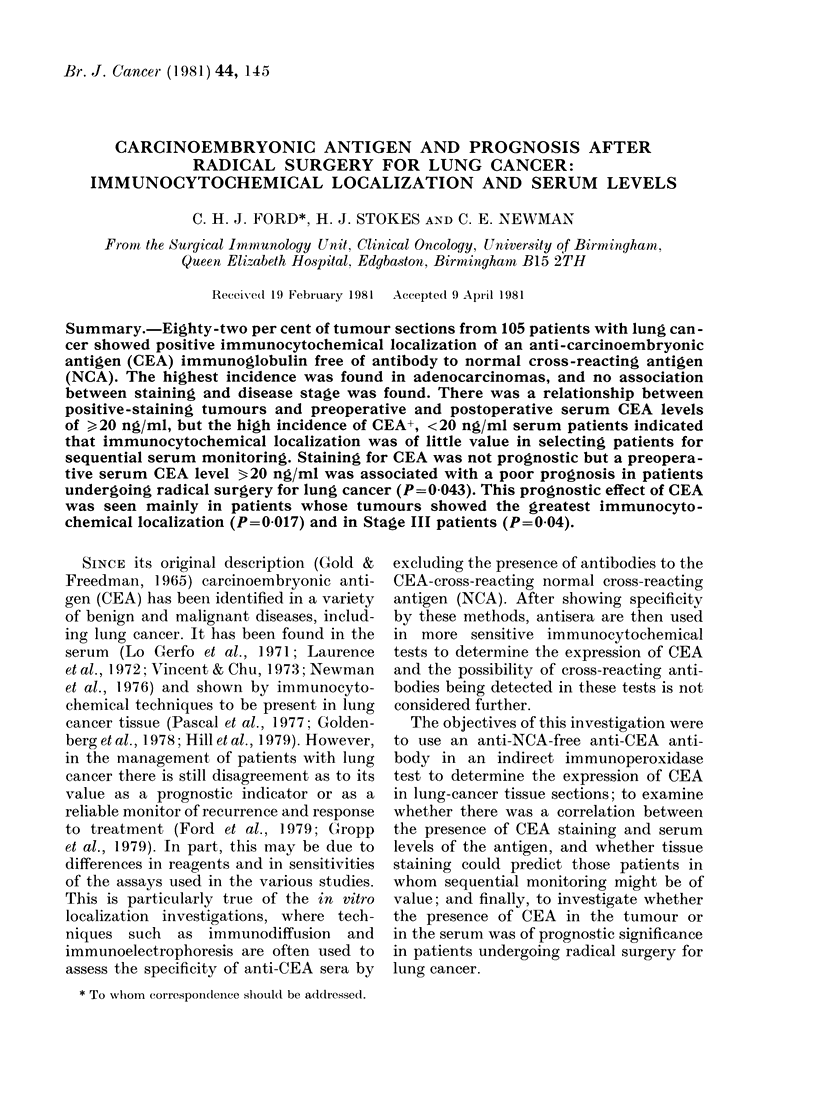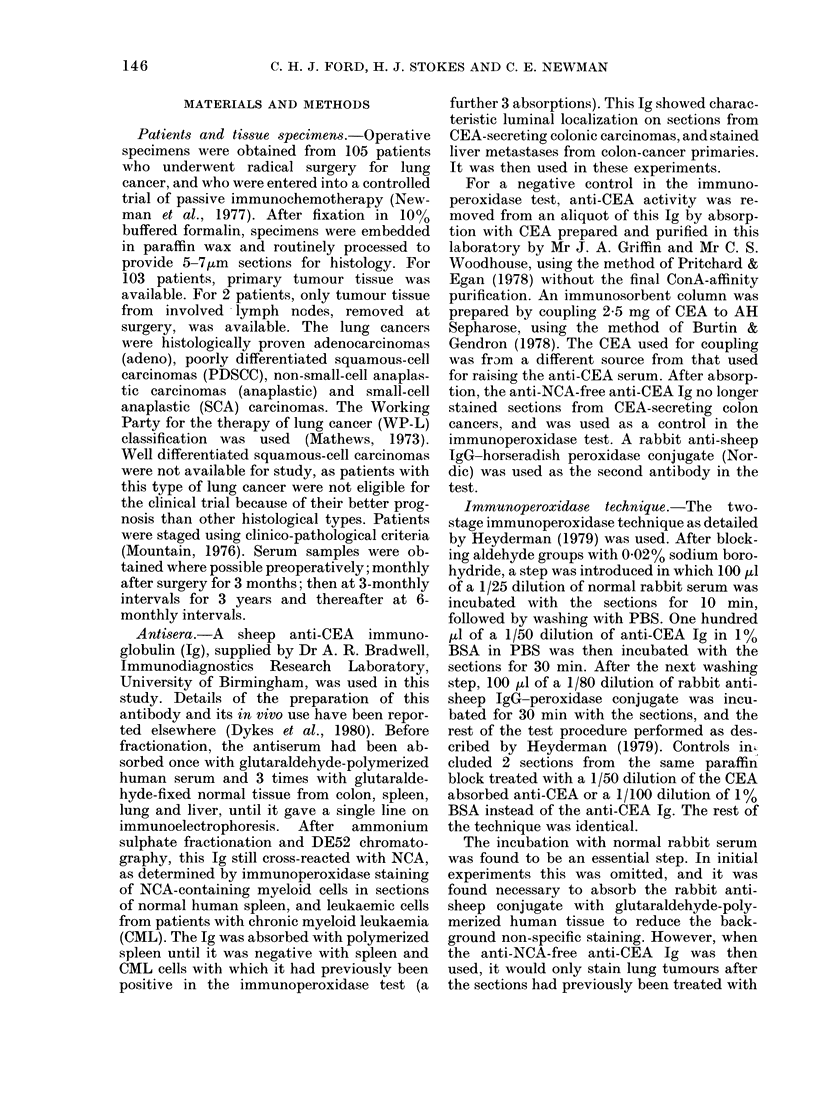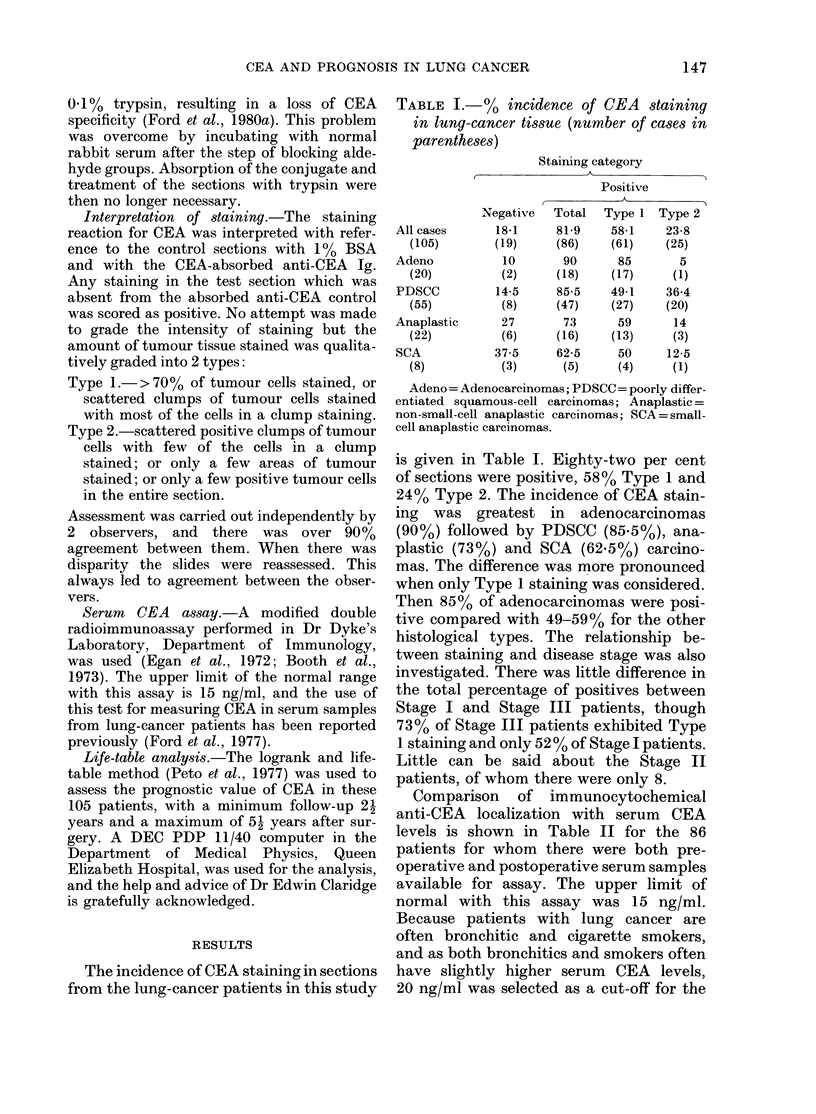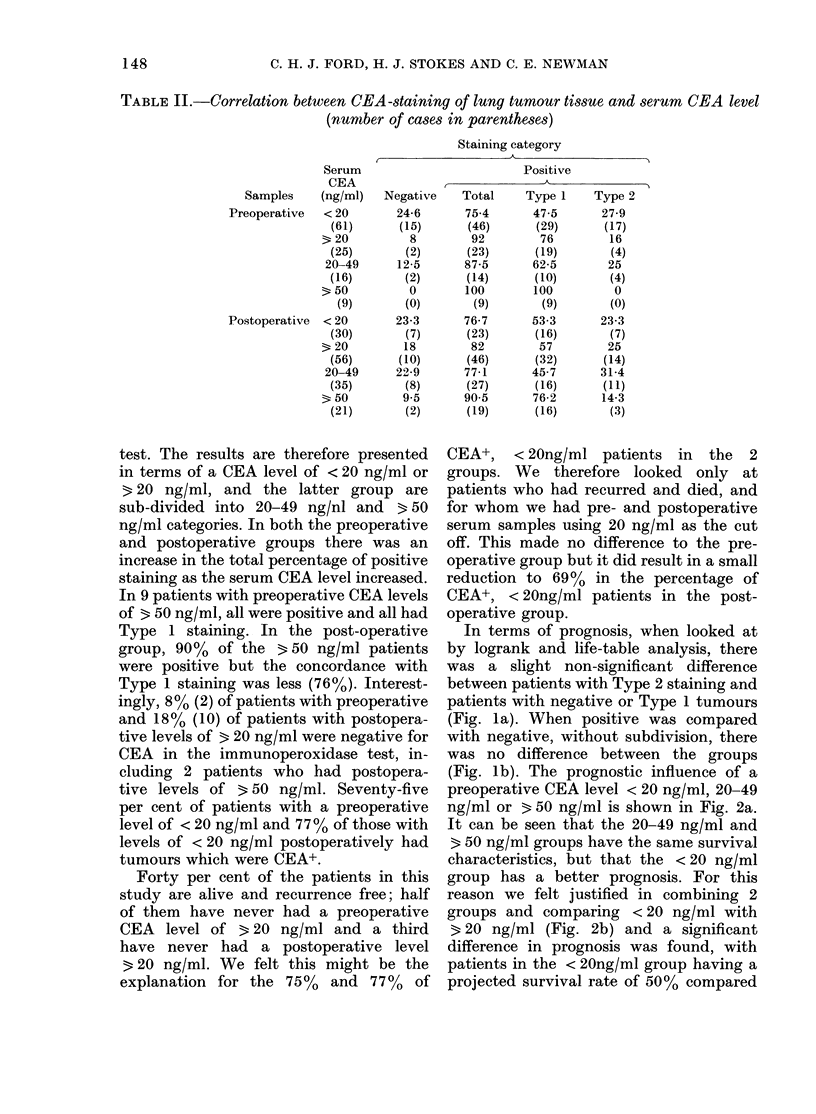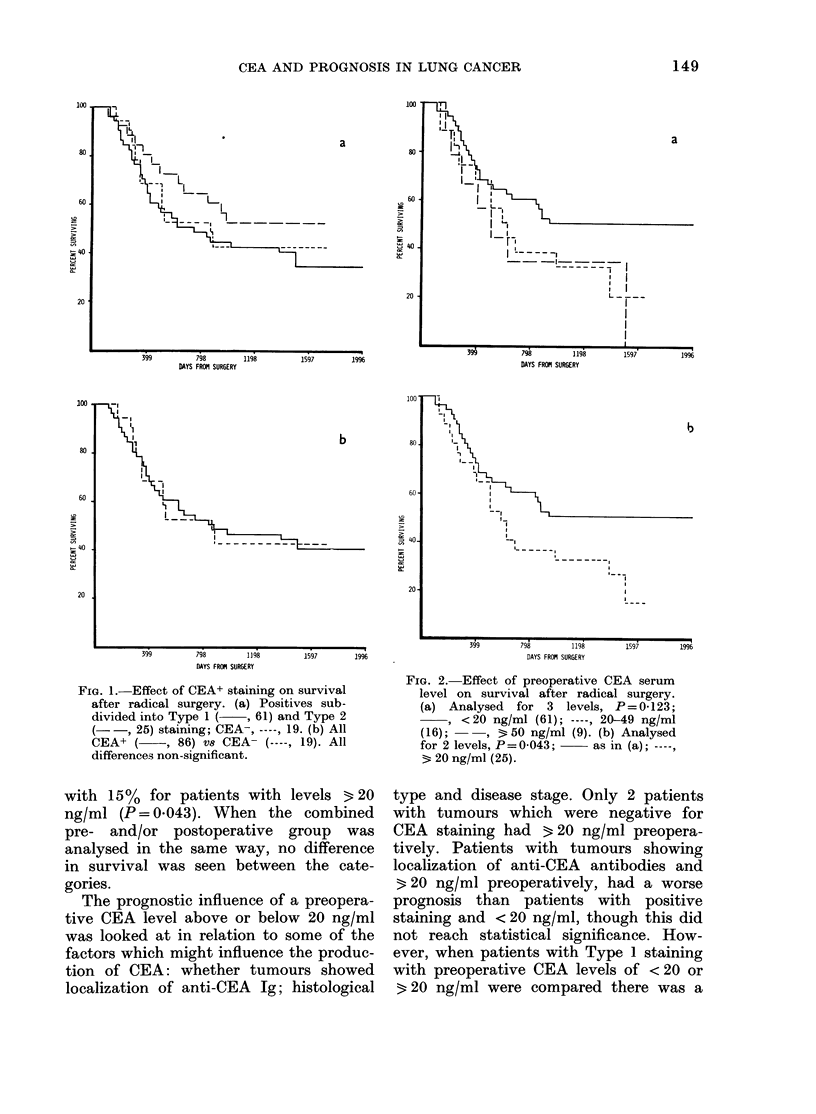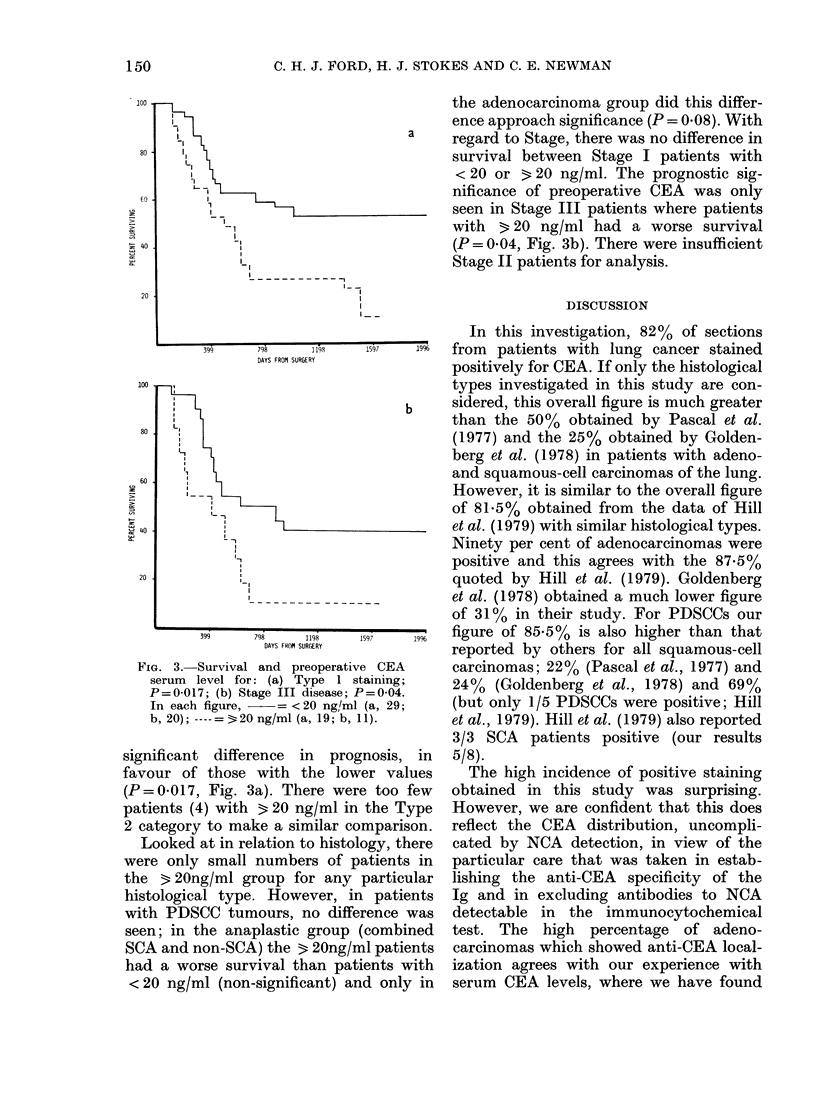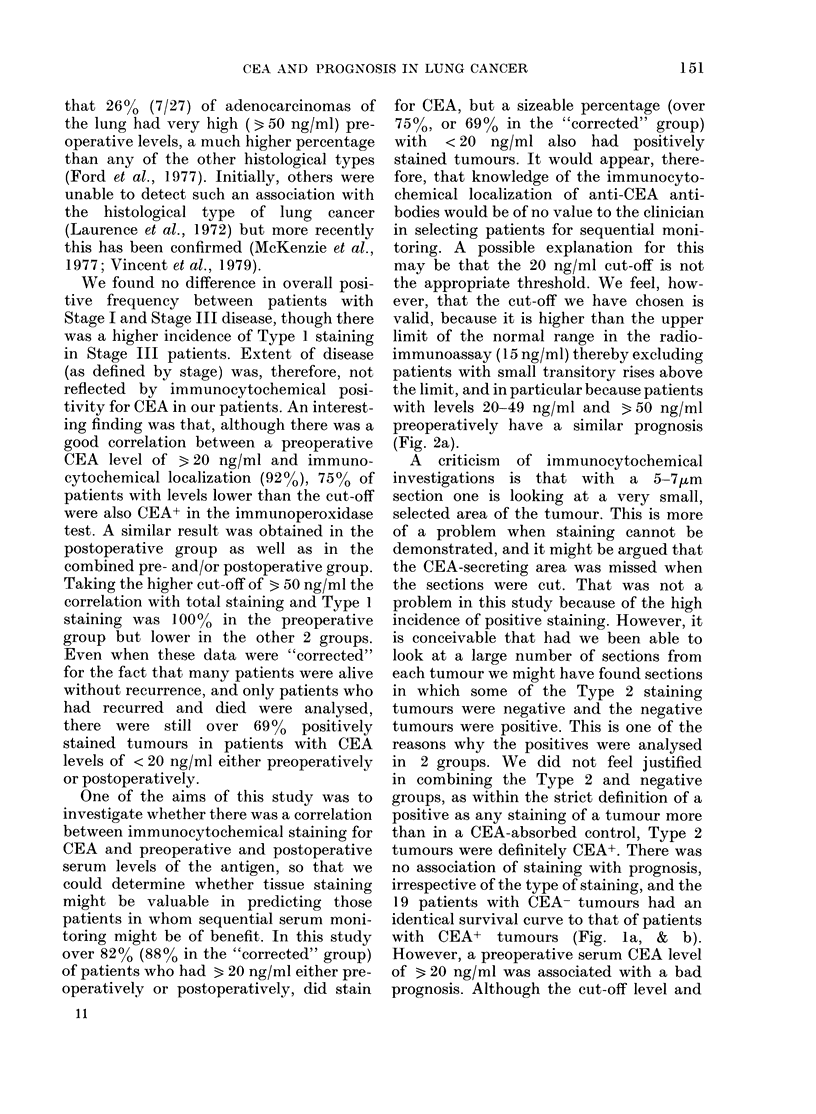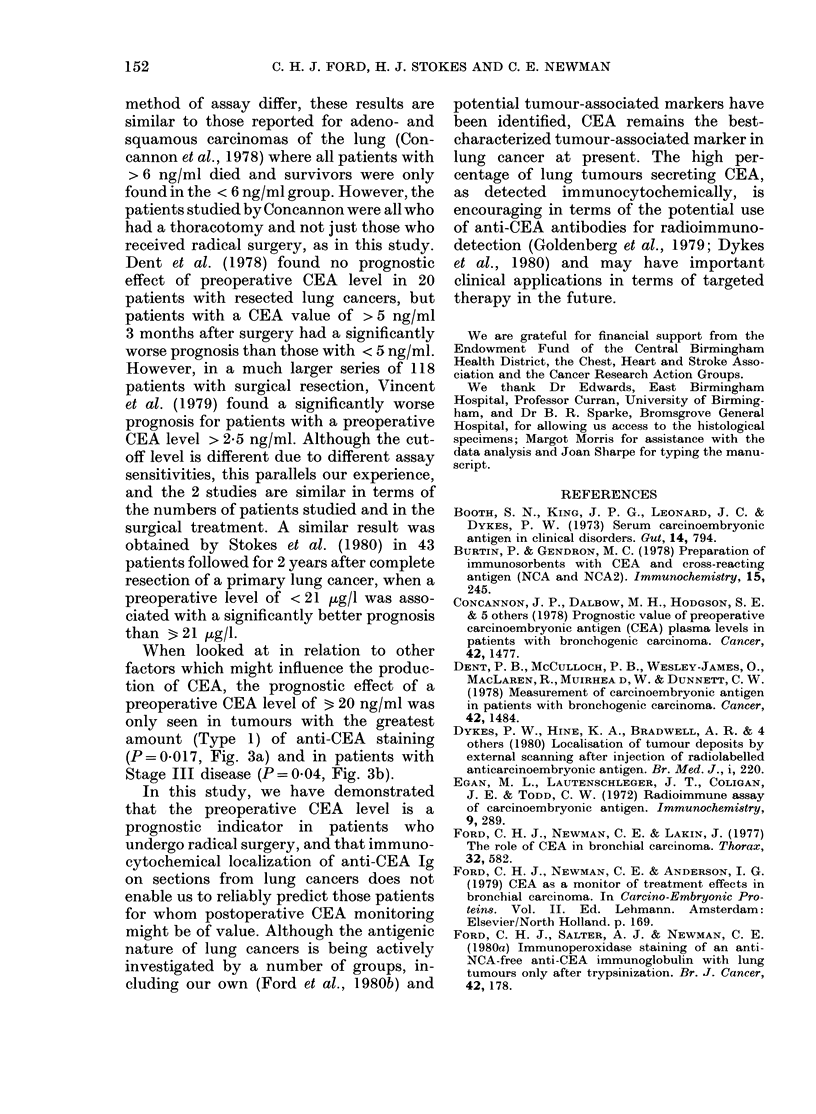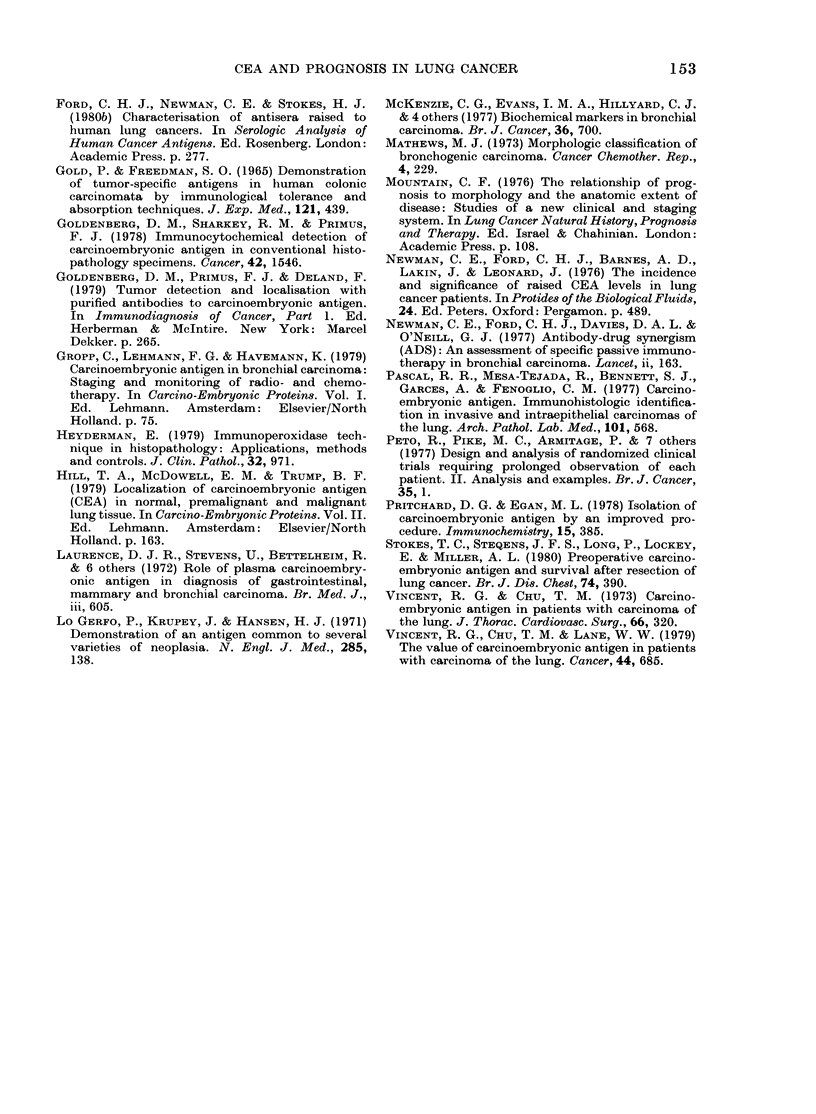# Carcinoembryonic antigen and prognosis after radical surgery for lung cancer: immunocytochemical localization and serum levels.

**DOI:** 10.1038/bjc.1981.164

**Published:** 1981-08

**Authors:** C. H. Ford, H. J. Stokes, C. E. Newman

## Abstract

Eighty-two per cent of tumour sections from 105 patients with lung cancer showed positive immunocytochemical localization of an anti-carcinoembryonic antigen (CEA) immunoglobulin free of antibody to normal cross-reacting antigen (NCA). The highest incidence was found in adenocarcinomas, and no association between staining and disease stage was found. There was a relationship between positive-staining tumours and preoperative and postoperative serum CEA levels of greater than or equal to 20 ng/ml, but the high incidence of CEA+, less than 20 ng/ml serum patients indicated that immunocytochemical localization was of little value in selecting patients for sequential serum monitoring. Staining for CEA was not prognostic but a preoperative serum CEA levels greater than or equal to 20 ng/ml was associated with a poor prognosis in patients undergoing radical surgery for lung cancer (P = 0.043). this prognostic effect of CEA was seen mainly in patients whose tumours showed the greatest immunocytochemical localization (P = 0.017) and in Stage III patients (P = 0.04).


*Br. J. Cancer* (1981) **44**, 145

### CARCINOEMBRYONIC ANTIGEN AND PROGNOSIS AFTER RADICAL SURGERY FOR LUNG CANCER: IMMUNOCYTOCHEMICAL LOCALIZATION AND SERUM LEVELS

C. H. J. FORD*, H. J. STOKES AND C. E. NEWMAN

*From the Surgical Immunology Unit, Clinical Oncology, University of Birmingham, Queen Elizabeth Hospital, Edgbaston, Birmingham B15 2TH*

Received 19 February 1981 Accepted 9 April 1981

**Summary.—Eighty-two per cent of tumour sections from 105 patients with lung cancer showed positive immunocytochemical localization of an anti-carcinoembryonic antigen (CEA) immunoglobulin free of antibody to normal cross-reacting antigen (NCA). The highest incidence was found in adenocarcinomas, and no association between staining and disease stage was found. There was a relationship between positive-staining tumours and preoperative and postoperative serum CEA levels of $\geqslant$20 ng/ml, but the high incidence of CEA$^+$, <20 ng/ml serum patients indicated that immunocytochemical localization was of little value in selecting patients for sequential serum monitoring. Staining for CEA was not prognostic but a preoperative serum CEA level $\geqslant$20 ng/ml was associated with a poor prognosis in patients undergoing radical surgery for lung cancer ($P=0{\cdot}043$). This prognostic effect of CEA was seen mainly in patients whose tumours showed the greatest immunocytochemical localization ($P=0{\cdot}017$) and in Stage III patients ($P=0{\cdot}04$).**

SINCE its original description (Gold & Freedman, 1965) carcinoembryonic antigen (CEA) has been identified in a variety of benign and malignant diseases, including lung cancer. It has been found in the serum (Lo Gerfo *et al.*, 1971; Laurence *et al.*, 1972; Vincent & Chu, 1973; Newman *et al.*, 1976) and shown by immunocytochemical techniques to be present in lung cancer tissue (Pascal *et al.*, 1977; Goldenberg *et al.*, 1978; Hill *et al.*, 1979). However, in the management of patients with lung cancer there is still disagreement as to its value as a prognostic indicator or as a reliable monitor of recurrence and response to treatment (Ford *et al.*, 1979; Gropp *et al.*, 1979). In part, this may be due to differences in reagents and in sensitivities of the assays used in the various studies. This is particularly true of the *in vitro* localization investigations, where techniques such as immunodiffusion and immunoelectrophoresis are often used to assess the specificity of anti-CEA sera by excluding the presence of antibodies to the CEA-cross-reacting normal cross-reacting antigen (NCA). After showing specificity by these methods, antisera are then used in more sensitive immunocytochemical tests to determine the expression of CEA and the possibility of cross-reacting antibodies being detected in these tests is not considered further.

The objectives of this investigation were to use an anti-NCA-free anti-CEA antibody in an indirect immunoperoxidase test to determine the expression of CEA in lung-cancer tissue sections; to examine whether there was a correlation between the presence of CEA staining and serum levels of the antigen, and whether tissue staining could predict those patients in whom sequential monitoring might be of value; and finally, to investigate whether the presence of CEA in the tumour or in the serum was of prognostic significance in patients undergoing radical surgery for lung cancer.

* To whom correspondence should be addressed.

### MATERIALS AND METHODS

*Patients and tissue specimens.*—Operative specimens were obtained from 105 patients who underwent radical surgery for lung cancer, and who were entered into a controlled trial of passive immunochemotherapy (Newman *et al.*, 1977). After fixation in 10% buffered formalin, specimens were embedded in paraffin wax and routinely processed to provide 5–7 $\mu$m sections for histology. For 103 patients, primary tumour tissue was available. For 2 patients, only tumour tissue from involved lymph nodes, removed at surgery, was available. The lung cancers were histologically proven adenocarcinomas (adeno), poorly differentiated squamous-cell carcinomas (PDSCC), non-small-cell anaplastic carcinomas (anaplastic) and small-cell anaplastic (SCA) carcinomas. The Working Party for the therapy of lung cancer (WP-L) classification was used (Mathews, 1973). Well differentiated squamous-cell carcinomas were not available for study, as patients with this type of lung cancer were not eligible for the clinical trial because of their better prognosis than other histological types. Patients were staged using clinico-pathological criteria (Mountain, 1976). Serum samples were obtained where possible preoperatively; monthly after surgery for 3 months; then at 3-monthly intervals for 3 years and thereafter at 6-monthly intervals.

*Antisera.*—A sheep anti-CEA immunoglobulin (Ig), supplied by Dr A. R. Bradwell, Immunodiagnostics Research Laboratory, University of Birmingham, was used in this study. Details of the preparation of this antibody and its *in vivo* use have been reported elsewhere (Dykes *et al.*, 1980). Before fractionation, the antiserum had been absorbed once with glutaraldehyde-polymerized human serum and 3 times with glutaraldehyde-fixed normal tissue from colon, spleen, lung and liver, until it gave a single line on immunoelectrophoresis. After ammonium sulphate fractionation and DE52 chromatography, this Ig still cross-reacted with NCA, as determined by immunoperoxidase staining of NCA-containing myeloid cells in sections of normal human spleen, and leukaemic cells from patients with chronic myeloid leukaemia (CML). The Ig was absorbed with polymerized spleen until it was negative with spleen and CML cells with which it had previously been positive in the immunoperoxidase test (a further 3 absorptions). This Ig showed characteristic luminal localization on sections from CEA-secreting colonic carcinomas, and stained liver metastases from colon-cancer primaries. It was then used in these experiments.

For a negative control in the immunoperoxidase test, anti-CEA activity was removed from an aliquot of this Ig by absorption with CEA prepared and purified in this laboratory by Mr J. A. Griffin and Mr C. S. Woodhouse, using the method of Pritchard & Egan (1978) without the final ConA-affinity purification. An immunosorbent column was prepared by coupling 2·5 mg of CEA to AH Sepharose, using the method of Burtin & Gendron (1978). The CEA used for coupling was from a different source from that used for raising the anti-CEA serum. After absorption, the anti-NCA-free anti-CEA Ig no longer stained sections from CEA-secreting colon cancers, and was used as a control in the immunoperoxidase test. A rabbit anti-sheep IgG–horseradish peroxidase conjugate (Nordic) was used as the second antibody in the test.

*Immunoperoxidase technique.*—The two-stage immunoperoxidase technique as detailed by Heyderman (1979) was used. After blocking aldehyde groups with 0·02% sodium borohydride, a step was introduced in which 100 $\mu$l of a 1/25 dilution of normal rabbit serum was incubated with the sections for 10 min, followed by washing with PBS. One hundred $\mu$l of a 1/50 dilution of anti-CEA Ig in 1% BSA in PBS was then incubated with the sections for 30 min. After the next washing step, 100 $\mu$l of a 1/80 dilution of rabbit anti-sheep IgG–peroxidase conjugate was incubated for 30 min with the sections, and the rest of the test procedure performed as described by Heyderman (1979). Controls included 2 sections from the same paraffin block treated with a 1/50 dilution of the CEA absorbed anti-CEA or a 1/100 dilution of 1% BSA instead of the anti-CEA Ig. The rest of the technique was identical.

The incubation with normal rabbit serum was found to be an essential step. In initial experiments this was omitted, and it was found necessary to absorb the rabbit anti-sheep conjugate with glutaraldehyde-polymerized human tissue to reduce the background non-specific staining. However, when the anti-NCA-free anti-CEA Ig was then used, it would only stain lung tumours after the sections had previously been treated with

0·1% trypsin, resulting in a loss of CEA specificity (Ford *et al.*, 1980*a*). This problem was overcome by incubating with normal rabbit serum after the step of blocking aldehyde groups. Absorption of the conjugate and treatment of the sections with trypsin were then no longer necessary.

*Interpretation of staining.*—The staining reaction for CEA was interpreted with reference to the control sections with 1% BSA and with the CEA-absorbed anti-CEA Ig. Any staining in the test section which was absent from the absorbed anti-CEA control was scored as positive. No attempt was made to grade the intensity of staining but the amount of tumour tissue stained was qualitatively graded into 2 types:

Type 1.—> 70% of tumour cells stained, or scattered clumps of tumour cells stained with most of the cells in a clump staining.

Type 2.—scattered positive clumps of tumour cells with few of the cells in a clump stained; or only a few areas of tumour stained; or only a few positive tumour cells in the entire section.

Assessment was carried out independently by 2 observers, and there was over 90% agreement between them. When there was disparity the slides were reassessed. This always led to agreement between the observers.

*Serum CEA assay.*—A modified double radioimmunoassay performed in Dr Dyke's Laboratory, Department of Immunology, was used (Egan *et al.*, 1972; Booth *et al.*, 1973). The upper limit of the normal range with this assay is 15 ng/ml, and the use of this test for measuring CEA in serum samples from lung-cancer patients has been reported previously (Ford *et al.*, 1977).

*Life-table analysis.*—The logrank and life-table method (Peto *et al.*, 1977) was used to assess the prognostic value of CEA in these 105 patients, with a minimum follow-up 2½ years and a maximum of 5½ years after surgery. A DEC PDP 11/40 computer in the Department of Medical Physics, Queen Elizabeth Hospital, was used for the analysis, and the help and advice of Dr Edwin Claridge is gratefully acknowledged.

### RESULTS

The incidence of CEA staining in sections from the lung-cancer patients in this study is given in Table I. Eighty-two per cent of sections were positive, 58% Type 1 and 24% Type 2. The incidence of CEA staining was greatest in adenocarcinomas (90%) followed by PDSCC (85·5%), anaplastic (73%) and SCA (62·5%) carcinomas. The difference was more pronounced when only Type 1 staining was considered. Then 85% of adenocarcinomas were positive compared with 49–59% for the other histological types. The relationship between staining and disease stage was also investigated. There was little difference in the total percentage of positives between Stage I and Stage III patients, though 73% of Stage III patients exhibited Type 1 staining and only 52% of Stage I patients. Little can be said about the Stage II patients, of whom there were only 8.

TABLE I.—*% incidence of CEA staining in lung-cancer tissue (number of cases in parentheses)*

| | Staining category | | | |
|---|---|---|---|---|
| | | Positive | | |
| | Negative | Total | Type 1 | Type 2 |
| All cases (105) | 18·1 (19) | 81·9 (86) | 58·1 (61) | 23·8 (25) |
| Adeno (20) | 10 (2) | 90 (18) | 85 (17) | 5 (1) |
| PDSCC (55) | 14·5 (8) | 85·5 (47) | 49·1 (27) | 36·4 (20) |
| Anaplastic (22) | 27 (6) | 73 (16) | 59 (13) | 14 (3) |
| SCA (8) | 37·5 (3) | 62·5 (5) | 50 (4) | 12·5 (1) |

Adeno = Adenocarcinomas; PDSCC = poorly differentiated squamous-cell carcinomas; Anaplastic = non-small-cell anaplastic carcinomas; SCA = small-cell anaplastic carcinomas.

Comparison of immunocytochemical anti-CEA localization with serum CEA levels is shown in Table II for the 86 patients for whom there were both preoperative and postoperative serum samples available for assay. The upper limit of normal with this assay was 15 ng/ml. Because patients with lung cancer are often bronchitic and cigarette smokers, and as both bronchitics and smokers often have slightly higher serum CEA levels, 20 ng/ml was selected as a cut-off for the

TABLE II.—*Correlation between CEA-staining of lung tumour tissue and serum CEA level (number of cases in parentheses)*

| Samples | Serum CEA (ng/ml) | Staining category: Negative | Positive: Total | Type 1 | Type 2 |
|---|---|---|---|---|---|
| Preoperative | < 20 | 24·6 | 75·4 | 47·5 | 27·9 |
| | (61) | (15) | (46) | (29) | (17) |
| | ≥ 20 | 8 | 92 | 76 | 16 |
| | (25) | (2) | (23) | (19) | (4) |
| | 20–49 | 12·5 | 87·5 | 62·5 | 25 |
| | (16) | (2) | (14) | (10) | (4) |
| | ≥ 50 | 0 | 100 | 100 | 0 |
| | (9) | (0) | (9) | (9) | (0) |
| Postoperative | < 20 | 23·3 | 76·7 | 53·3 | 23·3 |
| | (30) | (7) | (23) | (16) | (7) |
| | ≥ 20 | 18 | 82 | 57 | 25 |
| | (56) | (10) | (46) | (32) | (14) |
| | 20–49 | 22·9 | 77·1 | 45·7 | 31·4 |
| | (35) | (8) | (27) | (16) | (11) |
| | ≥ 50 | 9·5 | 90·5 | 76·2 | 14·3 |
| | (21) | (2) | (19) | (16) | (3) |

test. The results are therefore presented in terms of a CEA level of < 20 ng/ml or ≥ 20 ng/ml, and the latter group are sub-divided into 20–49 ng/nl and ≥ 50 ng/ml categories. In both the preoperative and postoperative groups there was an increase in the total percentage of positive staining as the serum CEA level increased. In 9 patients with preoperative CEA levels of ≥ 50 ng/ml, all were positive and all had Type 1 staining. In the post-operative group, 90% of the ≥ 50 ng/ml patients were positive but the concordance with Type 1 staining was less (76%). Interestingly, 8% (2) of patients with preoperative and 18% (10) of patients with postoperative levels of ≥ 20 ng/ml were negative for CEA in the immunoperoxidase test, including 2 patients who had postoperative levels of ≥ 50 ng/ml. Seventy-five per cent of patients with a preoperative level of < 20 ng/ml and 77% of those with levels of < 20 ng/ml postoperatively had tumours which were $CEA^+$.

Forty per cent of the patients in this study are alive and recurrence free; half of them have never had a preoperative CEA level of ≥ 20 ng/ml and a third have never had a postoperative level ≥ 20 ng/ml. We felt this might be the explanation for the 75% and 77% of $CEA^+$, < 20ng/ml patients in the 2 groups. We therefore looked only at patients who had recurred and died, and for whom we had pre- and postoperative serum samples using 20 ng/ml as the cut off. This made no difference to the preoperative group but it did result in a small reduction to 69% in the percentage of $CEA^+$, < 20ng/ml patients in the postoperative group.

In terms of prognosis, when looked at by logrank and life-table analysis, there was a slight non-significant difference between patients with Type 2 staining and patients with negative or Type 1 tumours (Fig. 1a). When positive was compared with negative, without subdivision, there was no difference between the groups (Fig. 1b). The prognostic influence of a preoperative CEA level < 20 ng/ml, 20–49 ng/ml or ≥ 50 ng/ml is shown in Fig. 2a. It can be seen that the 20–49 ng/ml and ≥ 50 ng/ml groups have the same survival characteristics, but that the < 20 ng/ml group has a better prognosis. For this reason we felt justified in combining 2 groups and comparing < 20 ng/ml with ≥ 20 ng/ml (Fig. 2b) and a significant difference in prognosis was found, with patients in the < 20ng/ml group having a projected survival rate of 50% compared

FIG. 1.—Effect of CEA+ staining on survival after radical surgery. (a) Positives subdivided into Type 1 (——, 61) and Type 2 (— —, 25) staining; CEA−, ----, 19. (b) All CEA+ (——, 86) *vs* CEA− (----, 19). All differences non-significant.

FIG. 2.—Effect of preoperative CEA serum level on survival after radical surgery. (a) Analysed for 3 levels, $P = 0{\cdot}123$; ——, $< 20$ ng/ml (61); ----, 20–49 ng/ml (16); — —, $\geqslant 50$ ng/ml (9). (b) Analysed for 2 levels, $P = 0{\cdot}043$; —— as in (a); ----, $\geqslant 20$ ng/ml (25).

with 15% for patients with levels $\geqslant 20$ ng/ml ($P = 0{\cdot}043$). When the combined pre- and/or postoperative group was analysed in the same way, no difference in survival was seen between the categories.

The prognostic influence of a preoperative CEA level above or below 20 ng/ml was looked at in relation to some of the factors which might influence the production of CEA: whether tumours showed localization of anti-CEA Ig; histological type and disease stage. Only 2 patients with tumours which were negative for CEA staining had $\geqslant 20$ ng/ml preoperatively. Patients with tumours showing localization of anti-CEA antibodies and $\geqslant 20$ ng/ml preoperatively, had a worse prognosis than patients with positive staining and $< 20$ ng/ml, though this did not reach statistical significance. However, when patients with Type 1 staining with preoperative CEA levels of $< 20$ or $\geqslant 20$ ng/ml were compared there was a

FIG. 3.—Survival and preoperative CEA serum level for: (a) Type 1 staining; $P=0\cdot017$; (b) Stage III disease; $P=0\cdot04$. In each figure, —— = < 20 ng/ml (a, 29; b, 20); ---- = ⩾20 ng/ml (a, 19; b, 11).

significant difference in prognosis, in favour of those with the lower values ($P=0\cdot017$, Fig. 3a). There were too few patients (4) with ⩾20 ng/ml in the Type 2 category to make a similar comparison.

Looked at in relation to histology, there were only small numbers of patients in the ⩾20ng/ml group for any particular histological type. However, in patients with PDSCC tumours, no difference was seen; in the anaplastic group (combined SCA and non-SCA) the ⩾20ng/ml patients had a worse survival than patients with < 20 ng/ml (non-significant) and only in the adenocarcinoma group did this difference approach significance ($P=0\cdot08$). With regard to Stage, there was no difference in survival between Stage I patients with < 20 or ⩾20 ng/ml. The prognostic significance of preoperative CEA was only seen in Stage III patients where patients with ⩾20 ng/ml had a worse survival ($P=0\cdot04$, Fig. 3b). There were insufficient Stage II patients for analysis.

### DISCUSSION

In this investigation, 82% of sections from patients with lung cancer stained positively for CEA. If only the histological types investigated in this study are considered, this overall figure is much greater than the 50% obtained by Pascal *et al.* (1977) and the 25% obtained by Goldenberg *et al.* (1978) in patients with adeno- and squamous-cell carcinomas of the lung. However, it is similar to the overall figure of 81·5% obtained from the data of Hill *et al.* (1979) with similar histological types. Ninety per cent of adenocarcinomas were positive and this agrees with the 87·5% quoted by Hill *et al.* (1979). Goldenberg *et al.* (1978) obtained a much lower figure of 31% in their study. For PDSCCs our figure of 85·5% is also higher than that reported by others for all squamous-cell carcinomas; 22% (Pascal *et al.*, 1977) and 24% (Goldenberg *et al.*, 1978) and 69% (but only 1/5 PDSCCs were positive; Hill *et al.*, 1979). Hill *et al.* (1979) also reported 3/3 SCA patients positive (our results 5/8).

The high incidence of positive staining obtained in this study was surprising. However, we are confident that this does reflect the CEA distribution, uncomplicated by NCA detection, in view of the particular care that was taken in establishing the anti-CEA specificity of the Ig and in excluding antibodies to NCA detectable in the immunocytochemical test. The high percentage of adenocarcinomas which showed anti-CEA localization agrees with our experience with serum CEA levels, where we have found that 26% (7/27) of adenocarcinomas of the lung had very high ($\geqslant 50$ ng/ml) preoperative levels, a much higher percentage than any of the other histological types (Ford *et al.*, 1977). Initially, others were unable to detect such an association with the histological type of lung cancer (Laurence *et al.*, 1972) but more recently this has been confirmed (McKenzie *et al.*, 1977; Vincent *et al.*, 1979).

We found no difference in overall positive frequency between patients with Stage I and Stage III disease, though there was a higher incidence of Type 1 staining in Stage III patients. Extent of disease (as defined by stage) was, therefore, not reflected by immunocytochemical positivity for CEA in our patients. An interesting finding was that, although there was a good correlation between a preoperative CEA level of $\geqslant 20$ ng/ml and immunocytochemical localization (92%), 75% of patients with levels lower than the cut-off were also $CEA^+$ in the immunoperoxidase test. A similar result was obtained in the postoperative group as well as in the combined pre- and/or postoperative group. Taking the higher cut-off of $\geqslant 50$ ng/ml the correlation with total staining and Type 1 staining was 100% in the preoperative group but lower in the other 2 groups. Even when these data were "corrected" for the fact that many patients were alive without recurrence, and only patients who had recurred and died were analysed, there were still over 69% positively stained tumours in patients with CEA levels of $<20$ ng/ml either preoperatively or postoperatively.

One of the aims of this study was to investigate whether there was a correlation between immunocytochemical staining for CEA and preoperative and postoperative serum levels of the antigen, so that we could determine whether tissue staining might be valuable in predicting those patients in whom sequential serum monitoring might be of benefit. In this study over 82% (88% in the "corrected" group) of patients who had $\geqslant 20$ ng/ml either preoperatively or postoperatively, did stain for CEA, but a sizeable percentage (over 75%, or 69% in the "corrected" group) with $<20$ ng/ml also had positively stained tumours. It would appear, therefore, that knowledge of the immunocytochemical localization of anti-CEA antibodies would be of no value to the clinician in selecting patients for sequential monitoring. A possible explanation for this may be that the 20 ng/ml cut-off is not the appropriate threshold. We feel, however, that the cut-off we have chosen is valid, because it is higher than the upper limit of the normal range in the radioimmunoassay (15 ng/ml) thereby excluding patients with small transitory rises above the limit, and in particular because patients with levels 20–49 ng/ml and $\geqslant 50$ ng/ml preoperatively have a similar prognosis (Fig. 2a).

A criticism of immunocytochemical investigations is that with a 5–7$\mu$m section one is looking at a very small, selected area of the tumour. This is more of a problem when staining cannot be demonstrated, and it might be argued that the CEA-secreting area was missed when the sections were cut. That was not a problem in this study because of the high incidence of positive staining. However, it is conceivable that had we been able to look at a large number of sections from each tumour we might have found sections in which some of the Type 2 staining tumours were negative and the negative tumours were positive. This is one of the reasons why the positives were analysed in 2 groups. We did not feel justified in combining the Type 2 and negative groups, as within the strict definition of a positive as any staining of a tumour more than in a CEA-absorbed control, Type 2 tumours were definitely $CEA^+$. There was no association of staining with prognosis, irrespective of the type of staining, and the 19 patients with $CEA^-$ tumours had an identical survival curve to that of patients with $CEA^+$ tumours (Fig. 1a, & b). However, a preoperative serum CEA level of $\geqslant 20$ ng/ml was associated with a bad prognosis. Although the cut-off level and method of assay differ, these results are similar to those reported for adeno- and squamous carcinomas of the lung (Concannon *et al.*, 1978) where all patients with $>6$ ng/ml died and survivors were only found in the $<6$ ng/ml group. However, the patients studied by Concannon were all who had a thoracotomy and not just those who received radical surgery, as in this study. Dent *et al.* (1978) found no prognostic effect of preoperative CEA level in 20 patients with resected lung cancers, but patients with a CEA value of $>5$ ng/ml 3 months after surgery had a significantly worse prognosis than those with $<5$ ng/ml. However, in a much larger series of 118 patients with surgical resection, Vincent *et al.* (1979) found a significantly worse prognosis for patients with a preoperative CEA level $>2{\cdot}5$ ng/ml. Although the cut-off level is different due to different assay sensitivities, this parallels our experience, and the 2 studies are similar in terms of the numbers of patients studied and in the surgical treatment. A similar result was obtained by Stokes *et al.* (1980) in 43 patients followed for 2 years after complete resection of a primary lung cancer, when a preoperative level of $<21$ $\mu$g/l was associated with a significantly better prognosis than $\geqslant 21$ $\mu$g/l.

When looked at in relation to other factors which might influence the production of CEA, the prognostic effect of a preoperative CEA level of $\geqslant 20$ ng/ml was only seen in tumours with the greatest amount (Type 1) of anti-CEA staining ($P=0{\cdot}017$, Fig. 3a) and in patients with Stage III disease ($P=0{\cdot}04$, Fig. 3b).

In this study, we have demonstrated that the preoperative CEA level is a prognostic indicator in patients who undergo radical surgery, and that immunocytochemical localization of anti-CEA Ig on sections from lung cancers does not enable us to reliably predict those patients for whom postoperative CEA monitoring might be of value. Although the antigenic nature of lung cancers is being actively investigated by a number of groups, including our own (Ford *et al.*, 1980*b*) and potential tumour-associated markers have been identified, CEA remains the best-characterized tumour-associated marker in lung cancer at present. The high percentage of lung tumours secreting CEA, as detected immunocytochemically, is encouraging in terms of the potential use of anti-CEA antibodies for radioimmunodetection (Goldenberg *et al.*, 1979; Dykes *et al.*, 1980) and may have important clinical applications in terms of targeted therapy in the future.

We are grateful for financial support from the Endowment Fund of the Central Birmingham Health District, the Chest, Heart and Stroke Association and the Cancer Research Action Groups.

We thank Dr Edwards, East Birmingham Hospital, Professor Curran, University of Birmingham, and Dr B. R. Sparke, Bromsgrove General Hospital, for allowing us access to the histological specimens; Margot Morris for assistance with the data analysis and Joan Sharpe for typing the manuscript.

### REFERENCES

BOOTH, S. N., KING, J. P. G., LEONARD, J. C. & DYKES, P. W. (1973) Serum carcinoembryonic antigen in clinical disorders. *Gut*, **14**, 794.

BURTIN, P. & GENDRON, M. C. (1978) Preparation of immunosorbents with CEA and cross-reacting antigen (NCA and NCA2). *Immunochemistry*, **15**, 245.

CONCANNON, J. P., DALBOW, M. H., HODGSON, S. E. & 5 others (1978) Prognostic value of preoperative carcinoembryonic antigen (CEA) plasma levels in patients with bronchogenic carcinoma. *Cancer*, **42**, 1477.

DENT, P. B., MCCULLOCH, P. B., WESLEY-JAMES, O., MACLAREN, R., MUIRHEA D, W. & DUNNETT, C. W. (1978) Measurement of carcinoembryonic antigen in patients with bronchogenic carcinoma. *Cancer*, **42**, 1484.

DYKES, P. W., HINE, K. A., BRADWELL, A. R. & 4 others (1980) Localisation of tumour deposits by external scanning after injection of radiolabelled anticarcinoembryonic antigen. *Br. Med. J.*, i, 220.

EGAN, M. L., LAUTENSCHLEGER, J. T., COLIGAN, J. E. & TODD, C. W. (1972) Radioimmune assay of carcinoembryonic antigen. *Immunochemistry*, **9**, 289.

FORD, C. H. J., NEWMAN, C. E. & LAKIN, J. (1977) The role of CEA in bronchial carcinoma. *Thorax*, **32**, 582.

FORD, C. H. J., NEWMAN, C. E. & ANDERSON, I. G. (1979) CEA as a monitor of treatment effects in bronchial carcinoma. In *Carcino-Embryonic Proteins*. Vol. II. Ed. Lehmann. Amsterdam: Elsevier/North Holland. p. 169.

FORD, C. H. J., SALTER, A. J. & NEWMAN, C. E. (1980*a*) Immunoperoxidase staining of an anti-NCA-free anti-CEA immunoglobulin with lung tumours only after trypsinization. *Br. J. Cancer*, **42**, 178.

FORD, C. H. J., NEWMAN, C. E. & STOKES, H. J. (1980*b*) Characterisation of antisera raised to human lung cancers. In *Serologic Analysis of Human Cancer Antigens*. Ed. Rosenberg. London: Academic Press. p. 277.

GOLD, P. & FREEDMAN, S. O. (1965) Demonstration of tumor-specific antigens in human colonic carcinomata by immunological tolerance and absorption techniques. *J. Exp. Med.*, **121**, 439.

GOLDENBERG, D. M., SHARKEY, R. M. & PRIMUS, F. J. (1978) Immunocytochemical detection of carcinoembryonic antigen in conventional histopathology specimens. *Cancer*, **42**, 1546.

GOLDENBERG, D. M., PRIMUS, F. J. & DELAND, F. (1979) Tumor detection and localisation with purified antibodies to carcinoembryonic antigen. In *Immunodiagnosis of Cancer, Part* 1. Ed. Herberman & McIntire. New York: Marcel Dekker. p. 265.

GROPP, C., LEHMANN, F. G. & HAVEMANN, K. (1979) Carcinoembryonic antigen in bronchial carcinoma: Staging and monitoring of radio- and chemotherapy. In *Carcino-Embryonic Proteins*. Vol. I. Ed. Lehmann. Amsterdam: Elsevier/North Holland. p. 75.

HEYDERMAN, E. (1979) Immunoperoxidase technique in histopathology: Applications, methods and controls. *J. Clin. Pathol.*, **32**, 971.

HILL, T. A., McDOWELL, E. M. & TRUMP, B. F. (1979) Localization of carcinoembryonic antigen (CEA) in normal, premalignant and malignant lung tissue. In *Carcino-Embryonic Proteins*. Vol. II. Ed. Lehmann. Amsterdam: Elsevier/North Holland. p. 163.

LAURENCE, D. J. R., STEVENS, U., BETTELHEIM, R. & 6 others (1972) Role of plasma carcinoembryonic antigen in diagnosis of gastrointestinal, mammary and bronchial carcinoma. *Br. Med. J.*, iii, 605.

LO GERFO, P., KRUPEY, J. & HANSEN, H. J. (1971) Demonstration of an antigen common to several varieties of neoplasia. *N. Engl. J. Med.*, **285**, 138.

McKENZIE, C. G., EVANS, I. M. A., HILLYARD, C. J. & 4 others (1977) Biochemical markers in bronchial carcinoma. *Br. J. Cancer*, **36**, 700.

MATHEWS, M. J. (1973) Morphologic classification of bronchogenic carcinoma. *Cancer Chemother. Rep.*, **4**, 229.

MOUNTAIN, C. F. (1976) The relationship of prognosis to morphology and the anatomic extent of disease: Studies of a new clinical and staging system. In *Lung Cancer Natural History, Prognosis and Therapy*. Ed. Israel & Chahinian. London: Academic Press. p. 108.

NEWMAN, C. E., FORD, C. H. J., BARNES, A. D., LAKIN, J. & LEONARD, J. (1976) The incidence and significance of raised CEA levels in lung cancer patients. In *Protides of the Biological Fluids*, **24**. Ed. Peters. Oxford: Pergamon. p. 489.

NEWMAN, C. E., FORD, C. H. J., DAVIES, D. A. L. & O'NEILL, G. J. (1977) Antibody-drug synergism (ADS): An assessment of specific passive immunotherapy in bronchial carcinoma. *Lancet*, ii, 163.

PASCAL, R. R., MESA-TEJADA, R., BENNETT, S. J., GARCES, A. & FENOGLIO, C. M. (1977) Carcinoembryonic antigen. Immunohistologic identification in invasive and intraepithelial carcinomas of the lung. *Arch. Pathol. Lab. Med.*, **101**, 568.

PETO, R., PIKE, M. C., ARMITAGE, P. & 7 others (1977) Design and analysis of randomized clinical trials requiring prolonged observation of each patient. II. Analysis and examples. *Br. J. Cancer*, **35**, 1.

PRITCHARD, D. G. & EGAN, M. L. (1978) Isolation of carcinoembryonic antigen by an improved procedure. *Immunochemistry*, **15**, 385.

STOKES, T. C., STEQENS, J. F. S., LONG, P., LOCKEY, E. & MILLER, A. L. (1980) Preoperative carcinoembryonic antigen and survival after resection of lung cancer. *Br. J. Dis. Chest*, **74**, 390.

VINCENT, R. G. & CHU, T. M. (1973) Carcinoembryonic antigen in patients with carcinoma of the lung. *J. Thorac. Cardiovasc. Surg.*, **66**, 320.

VINCENT, R. G., CHU, T. M. & LANE, W. W. (1979) The value of carcinoembryonic antigen in patients with carcinoma of the lung. *Cancer*, **44**, 685.